# Looking Beyond the Screen to Study the Technology Use of Older People Experiencing Cognitive Concerns

**DOI:** 10.1145/3772318.3790526

**Published:** 2026-04-13

**Authors:** Ruipu Hu, Eun Kyoung Choe, Amanda Lazar

**Affiliations:** College of Information University of Maryland College Park, Maryland, USA; College of Information University of Maryland College Park, Maryland, USA; College of Information University of Maryland College Park, Maryland, USA

**Keywords:** Videoconferencing, Older Adults, Cognitive Concerns, Design for Aging, Accessibility

## Abstract

Research with older adults has hinted at the ways that elements beyond the interface play a role in technology use, including videoconferencing. To further understand the range of materials and resources involved, we studied videoconferencing use by ten older individuals with cognitive concerns in a week-long study of interviews, observations, and a modified diary study. Our analysis identified that objects extending beyond software and hardware play a role in videoconferencing, including paper-based objects, personal items, and objects in the built environment. These objects support participants by externalizing information difficult to recall, distributing cognitive effort across time, and lowering cognitive load through their spatial placement and affordances. These insights point to opportunities for researchers working with older people to focus on the work happening outside of today’s interfaces. We also discuss how the lens of distributed cognition could help us design better technologies to support age-related cognitive impairment.

## 1. Introduction

Human-Computer Interaction research with older people typically focuses on *interactions with a technology*. We gather information about how older people are currently using a wide range of technologies or study how they adopt or choose not to adopt new technologies [45]. This focus has yielded rich insights, including how interfaces can be redesigned to better support older people.

And yet, elements beyond the interface have been making themselves known. Walls and outlets, paper-based technology manuals, and pets impact older people’s use of voice assistants [72]. Studies of videoconferencing with older adults, the focus area of our study, have a notable presence of salient elements outside of the technology interface. For example, an analysis of digital social activities of older adults during the pandemic found packages of materials, such as quilting materials, traveling through the mail to members of videoconferencing activity groups [89]. Another study features participants discussing the merits of meal trolleys versus bedroom furniture for holding up a tablet [36]. Non-technological objects appear to be playing a role in videoconferencing, but have largely appeared as fringe findings in work to date with older adults.

Videoconferencing is a useful application with which to pinpoint the use of elements beyond the interface. As opposed to, for example, texting on a phone or checking social media, a videoconference often has a defined start and end time and is arranged in advance, yielding the opportunity to observe use and for the researcher to directly gather participant reflections in the moment.

With our focus on the role objects play in technology use, we chose to engage with participants with cognitive concerns, a term we use in this paper that include mild cognitive impairment (MCI) as well as subjective cognitive decline (SCD), a “self-reported experience of worsening or more frequent confusion or memory loss.” Given the importance of objects and embodied interaction for people with more significant cognitive impairment, and the ways that elements beyond the interface have been appearing in studies of videoconferencing with older adults [22, 46, 47, 56, 66, 86], we engaged ten individuals with cognitive concerns in a week-long study. We collected data through interviews, an observation of videoconferencing use, and a modified diary study to capture relevant objects. We chose this method based on past work that shows that non-verbal data is an effective way of accessing data of people with cognitive impairments [96]. Our study answers the following research questions:

**RQ1:** What types of objects do participants with cognitive concerns interact with in the context of a technology activity (specifically, a videoconference)?

**RQ2:** How do objects support videoconferencing for participants with cognitive concerns?

Operating with the definition of an object as something you can see, hear, smell, touch, or taste, we found a range of objects from participants’ daily lives involved in videoconferencing (RQ1). As might be expected, this included hardware devices as well as videoconferencing software or web platforms. Participants used many other kinds of software and hardware outside of the videoconferencing technology. In addition, they engaged with a wide range of objects that would not typically be considered in the purview of a user-centered design lens. This included paper-based objects, personal items, objects in the built environment, and other (e.g., pets) (4.1).

To answer RQ2, how objects support videoconferencing, we found that objects support cognition through holding information that is important for videoconferencing but difficult to recall, such as semantic and time-related information (4.2). Objects distribute effort across time, through leveraging optimal cognitive functioning times ahead of a call to prepare and short-term memory during a call to document things for later (4.3). And, participants leverage different qualities of objects arranged in their space to keep their focus on the videoconference (4.4).

Our work contributes to research supporting aging in place by pointing to design opportunities outside of the interface. We also discuss how the lens of distributed cognition could help us design better technologies to support cognitive functioning. These contributions add to the growing body of literature understanding how barriers to technologies, including videoconferencing applications, can be reduced [16, 17, 62, 67, 81, 83, 97].

## 2 Related Work

Below, we describe prior research on objects in studies with older adults and work examining their use of videoconferencing technology.

### 2.1 Objects in Research with Older Adults

Researchers have long recognized the importance of objects in HCI and ubiquitous computing research. Early visions of ubiquitous computing including physical and tangible objects as part of everyday computing [1, 34]. For example, Ishii and Ullmer [34] proposed tangible objects as a seamless connection with cyberspace. They proposed categories for everyday tangible interfaces, including graspable items like cards and books, as well as ”ambient media” objects such as sound, light, and airflow as background interfaces at the “periphery of human perception” [34].

An attention to objects has also existed in research with older people. The significance the objects hold has been leveraged to support aging in place and fostering positive aging experiences [5, 9, 19, 28, 52, 84, 98]. Researchers are designing technology to support social interactions with others around meaning-producing objects [10, 31, 55, 77, 84] such as photographs [55] and water kettles [10, 31].

Beyond utilizing objects to support interaction, researchers are seeking to capture the ways objects are used in the house [5, 23, 39, 41, 99, 100]. Whether a front door is open or closed, or the stove is on or off [54], can make a difference in someone’s ability to live independently [54, 82, 91]. As such, researchers are designing systems that capture information about objects (e.g., stove left on [54]) and then prompt people, including older adults, accordingly [50]. Interactions with objects such as the stove and the door can also yield useful information about someone’s functioning, and other researchers [48, 70] seek to understand interactions with objects in terms of the signals they give about health and wellbeing.

Alongside these explorations, formative studies on objects have been important to surface needs that had not been articulated before and lay the groundwork for future interventions. Kuoppamäki et al. studied recordings of six older adults preparing a meal in the kitchen, attending to the interactions with and organization of objects such as knives and ingredients, to improve cooking workflows and support older adults’ ability to continue living independently at home [41]. Also in the kitchen context, Chan et al. [13] explored how context-aware systems might scaffold everyday cooking tasks and interactions in the kitchen, suggesting “using sensors connected to objects to guide them in placing items back where they belong” to support memory challenges of older adults. Our study complements this past work by examining the types of objects involved in videoconferencing and the needs they may reveal for people with cognitive concerns.

Objects appear particularly important for people with memory concerns. In advanced dementia, prior research has identified the ways that objects offer support, partly due to the ways they shift interaction from verbal communication to embodied forms that remain accessible far into the condition [22, 46, 47, 56, 66, 86]. When appropriately engaged, objects can serve as critical external cognitive supports for individuals experiencing memory concerns. Simple artifacts such as notes [29], reminder devices and systems [11, 78] are tangible tools that can scaffold memory and aid daily functioning. Thus, studying, instrumenting, and tracking the objects that older adults interact with in their homes holds particular potential for people with memory and other cognitive concerns.

Overall, objects have become increasingly central to research on age-related and cognitive changes, as tools of identifying technological problems, informing meaningful design, and understanding everyday cognition. Yet, we still lack foundational knowledge about what kinds of objects participate in people’s technology activities and how these objects function within technology environments and cognitive processes.

### 2.2 Videoconferencing Technology and Older Adults

Past research has demonstrated that videoconferencing can support a wide range of activities for many, including older adults, such as engagement in diverse social [15, 29], health [44, 62, 97], and leisure activities [30]. Videoconferencing also provides benefits that in-person participation might not offer, such as reducing travel burdens and flexible access to activities from home for older adults with mobility limitations, such as older adult in aged care services [25, 101]. Focused on how videoconferencing can support virtual activities, these studies often seek to uncover older adults’ use through interviews [29, 36, 89]. Some of these past research has surfaced objects in the user’s immediate physical environment as relevant [36, 89] – as our research seeks to center material and digital artifacts and the role they play in videoconferencing, we opted for a week long study involving in-person interviews but also observations and a diary study all designed around making these objects more visible to participants and researchers.

Some studies have found that videoconferencing can be a beneficial medium for individuals with cognitive impairment to engage in activity [29, 30, 67, 89]. Some work has explored how videoconferencing platforms can aid in detecting cognitive concerns remotely [62, 97], while others have shown that videoconferencing can help older adults navigate changes related to cognitive abilities [29, 67, 102]. For example, videoconferencing has been used to facilitate social sharing within dementia communities [102], support continued online participation among people with mild cognitive impairment (MCI) ) [67], and enable individuals to adapt to new identities such as becoming dementia advocates by engaging in online discussion groups [29].

Importantly, engaging with videoconferencing is rarely limited to the digital interface alone. It can involve interactions with a range of physical artifacts, such as physical notes [15, 29, 30], assistive devices and accessibility tools such as prescription glasses [15] or a dedicated device for a video call to the ASL interpreter [103], as well as coordination with other people and environmental adjustments [51]. This complexity makes videoconferencing interactions rich and interesting, but also introduces significant challenges, particularly for older adults with cognitive concerns. Difficulties in managing multiple layers of interaction such as switching between physical notes and on-screen content, or maintaining orientation in a multitasking environment can create barriers to people’s cognitive abilities such as hindering their ability in attention, executive functioning and memory [29, 42, 51]. Our study shows how people with cognitive concerns engage with objects to overcome some of these difficulties, enabling them to reserve their cognitive capacity for the videoconference itself.

Past HCI research has tried to improve the videoconferencing experience with various approaches, including understanding the social dynamics of virtual meetings [29, 53, 69], designing tools that better support collaboration [2, 4, 58, 87], and creating interfaces that are easier to navigate for diverse user groups [20, 60, 64, 75, 80, 104]. In the field of ubiquitous computing, researchers have further explored how videoconferencing becomes interwoven into everyday environments across various domains such as education [14], creativity [24], and programming [105]. Some approaches augment interactions through peripheral displays and multimodal inputs to support more seamless experiences [18]. While progress has been made toward improving videoconferencing technologies, there remains room for greater attention to the experiences of older adults with cognitive impairments.

## 3 Methods

Below, we describe the study design, data collection procedures, recruitment strategies, and data analysis process. Procedures were approved by the university’s Institutional Review Board (IRB #1316631). Participants received financial compensation ($60 USD) for their involvement in the study.

### 3.1 Study Design and Data Collection

All participants provided informed consent on their own before participating in the study. Over the course of one week, each participant engaged in two in-person interviews, an in-person observation, and a modified diary study.

In **interview 1** (one-two hours), we first collected demographic information and a detailed understanding of participants’ everyday cognitive concerns (Appendix A1). To develop this interview protocol, our research team consulted with a health professional with expertise in working with older people with cognitive impairment (e.g., MCI, dementia and Alzheimer’s disease), Through his expertise, we were able to develop a protocol informed by established clinical frameworks describing common cognitive domains affected by aging, including memory, language, and spatial orientation. We iteratively refined the protocol through internal pilot testing with first and last authors and with feedback from the health professional. These pilots helped clarify question wording, adjust the order of topics, and increase the accessibility and comprehensibility for participants with different levels of cognitive concerns.

After collecting the demographic information and understanding of participants’ everyday cognitive concerns, the researcher introduced the study to participants and demonstrated use of the materials for the modified diary study. We asked participants to select a videoconference that they regularly engaged in for us to come observe during the week of the study. Sessions were audio recorded (11 hours in total).

The in-person **observation** (one-two hours) occurred midweek. We began our observation ten minutes prior and ended it ten minutes after participants engaged in videoconferencing in their usual locations. We recorded audio and video where consent was given, and collected field notes and photographs to focus on the objects with which participants interacted (14 hours observation in total).

**Interview 2** (one-two hours) occurred on the final day of the study. We conducted an unstructured interview where we asked participants to reflect on the objects they documented during the modified diary study. Participants were encouraged to share why certain objects were captured, how those objects supported their videoconferencing practices, and how they related to the cognitive concerns identified in Interview 1. We also followed up on our observations in cases where participants did not capture objects we had seen them use, inviting them to explain these choices or omissions. Sessions were audio recorded (15 hours in total).

**Figure 1:**
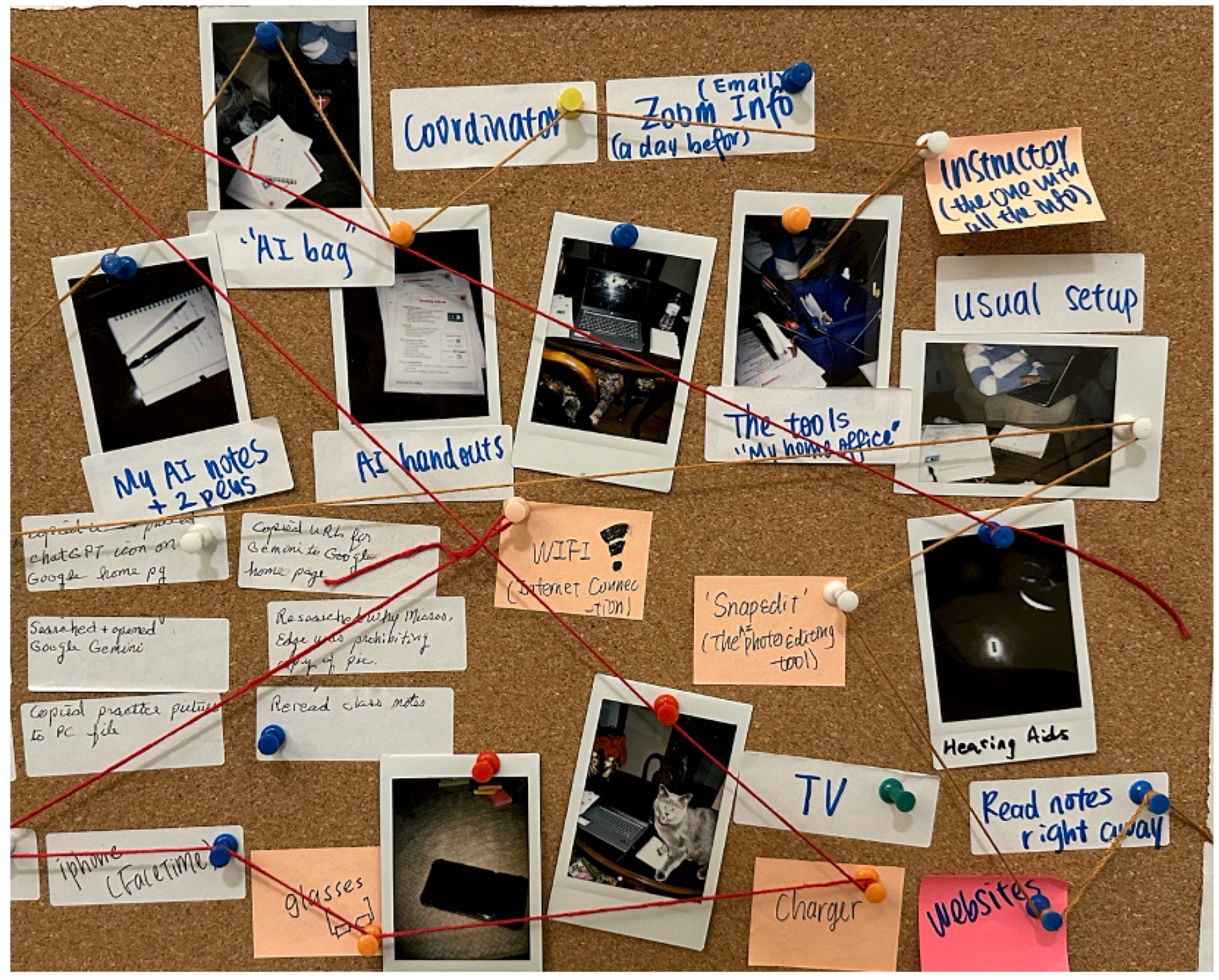
This image shows a part of Taylor’s corkboard, where she documented objects as part of our modified diary study.

The **modified diary study** (week-long data capture) had been introduced to participants in the first interview, and they filled it out during the week of the study. We prompted participants to capture and document objects they “*interacted with that were relevant to videoconferencing use*.” We listed categories to guide their documentation: the physical environment, engaged objects, people, tools, technologies and electronics, as well as personal and miscellaneous items. Participants used a Polaroid camera, sticky notes, label stickers, and markers to document their objects, and a corkboard, scissors, glue dots, and yarn to organize and display their captured items (see Figure 1 of one participant’s finished board). The ten participants created and titled a diverse set of diaries (Table 1). When participants requested assistance with documentation for accessibility reasons, the researcher did so on their behalf. The researcher discussed the board with participants during interview 2.

### 3.2 Recruitment and Participants

We had the following criteria for recruitment: participants had to 1) report cognitive concerns, which could include subjective cognitive decline (SCD)^2^ , mild cognitive impairment (MCI)^3^, or dementia^4^; 2) be aged 50 or over; 3) engage in videoconferencing at least once a week; and 4) reside within a distance that the researcher could travel to for in-person sessions. Participants also had to have the capacity to consent by themselves to the study in order to participate. We recruited a convenience sample from our local community and networks through word of mouth, personal contacts and flyers. Given that a member of our research team speaks Mandarin, we were able to include 6 participants who preferred to speak in Mandarin during interviews.

In total, we recruited nine participants with SCD, and one participant with MCI. The average age of ten participants is 71.9 (with one participant in their late 50s). Table 2 reports additional demographics including age range, race/ethnicity, and information about cognitive concerns.

### 3.3 Data Analysis

Our focus in this study is on objects involved in videoconferencing. We defined objects as things that are registered or interacted with through the human senses – objects can be felt, seen, or heard, or even smelled or tasted^5^. To surface objects from the data, the first author reviewed transcripts, diaries, observation notes, and images and videos from the observation and noted objects that: (1) participants captured during the diary study, (2) mentioned during interviews or observation sessions, and (3) that the researcher identified as related to videoconferencing. In total, we identified 281 objects. We engaged in affinity diagramming to generate a typology of objects (RQ1). In Miro (an online whiteboard and virtual workspace) [95], the first author placed digital sticky notes with objects, the quote where the object appeared, and the rationale for its inclusion based on one of the three criteria above. A unique note was created for every object mentioned by a participant. We inductively coded objects, clustering similar ones and labeling shared features. We iteratively refined and consolidated categories. After generating 57 initial categories, we moved to higher-level groupings tied to broader aspects of daily life – for example, “rooms,” “structural elements,” and “furniture” were combined under “built environment.” Our final categorization comprised six categories: hardware, software and web platforms, paper-based objects, personal items, built environment, and other.

To answer RQ2 (“How do objects support videoconferencing for participants with cognitive concerns?”), we followed the six steps of reflexive thematic analysis [6]. The first step started with first author familiarizing themselves with the data by listening to all audio recordings and verifying transcripts that had been automatically generated through Dovetail, a qualitative analysis platform [106]. The first author then began the second step of generating codes to label all collected data, including all interview transcripts, observation notes and other media such as images and videos. During this step, the first author created memos to document initial thoughts and observations, which were regularly discussed with the last author. We then inductively grouped initial codes into candidate themes, followed by several rounds of reviewing and refining these themes to clarify their meanings and identify similarities and differences. For example, a candidate theme identified by the first author was titled “difficulties in recalling information”. This third author pointed out that this coding approach implicitly places blame on people with cognitive impairments, which may exacerbate the deficit framing long existed in research history with this population. This prompted us to shift our attention back to initial codes and toward themes that emphasized object qualities such as “grabble” and the “temporality” of objects, rather than participant deficits.

We provide this example to illustrate why for this (reflexive thematic) analysis, inter-rater reliability (IRR) or forcing coder agreement is not appropriate or necessary [63]. When scholarly orientations aim to challenge hegemonic categories of knowledge, as in our effort to move away from deficit framings, inter-rater reliability can constrain interpretive diversity and inadvertently reproduce the very assumptions that the analysis seeks to unsettle.

As we reviewed our themes, we realized that distributed cognition [27, 33, 38] offered a lens that brought together the cognition, and affordances and temporality of objects in a more cohesive way: for example, in one iteration, we grouped “*support semantic recall*” and “*support temporal recall*” under “*Objects hold information*.”

Reflecting on how our own worldviews affected our data analysis, we have seen how older people with cognitive concerns can be “othered” and seen as lacking. We believe that people like the participants we worked with are not so different from other groups, though older people and people with MCI in particular are represented as such in much of the literature. Like we do, participants navigate complex technology and material environments, and make purposeful and intelligent decisions to address their circumstances. These beliefs likely led us to look for analytic perspectives that could show how the technology use that we observed makes sense in the context of how people think and remember through their environments.

Finally, reflecting on our choice of adopting distributed cognition, there are other theoretical lenses that could provide different perspectives on the data. Embodiment interaction [21] focusing on one’s body’s physical experiences like eye gaze, gestures, and body movements, has been highlighted in critical dementia scholarship but does not provide the most ideal language to answer questions such as RQ2 on how objects support people’s cognition in technology use. In a similar vein, situated action [79] can illuminate the complexity of the context and how people react to it, but has less detailed focus on people’s cognition – which is key in our study given the population with whom we are working.

We briefly reflect on the use, reliability, validity of our reflexive thematic analysis process. In reflexive thematic analysis and other interpretivist methods, reliability is not assessed via multiple coder agreement (e.g., inter-rater reliability), as the goal is not to summarize the data “accurately” or minimize subjectivity. Rather, by engaging with the steps of reflexive thematic analysis, “the aim is to provide a coherent and compelling *interpretation* of the data” (p848, [8]). In terms of validity, this is established through collaborative engagement of the research team, which occurred throughout the analysis process as described above [7].

### 3.4 Limitations

We lack data on social partners. Although the study design allowed participants to include a relevant partner (e.g., carer, family member, or friend), none chose to do so. Because this project focuses specifically on the technology use of individuals with cognitive impairments, our analysis centers on the individual experience. Researchers interested in the collaborative dimensions of videoconferencing should therefore be aware that our findings do not capture how interactions may be jointly coordinated with others. For example, it is possible that some of the preparation work of having the right object in place at the right time may have been facilitated by a caregiver or social partner, as has been found in past work about older people’s videoconferencing [36]. Since our analysis is focused on the ways that objects are used in ways we could observe or were described participants, we believe additional study of the role of others in these arrangements will complement but not negate our findings.

Also, there are many people with identities that are not represented in any of the study participants. In addition, while there was some diversity in terms of cognitive, visual, auditory, and motor abilities, our discussion of affordances does not fully acknowledge how “relational” the nature of these affordances are–“*A situation has an affordance for a particular agent*” [38]. This led to findings of interactions that may be more accessible for some, but less for others. For example, glanceable interfaces as described in our examples are not going to be accessible for Blind users. This should be kept in mind for drawing on these concepts.

Finally. the study did not focus on a single kind of videoconferencing activity. Future studies that focus on distinct types of videoconferencing activity may yield additional insights.

## 4 FINDINGS

Our findings detail how materials and resources in people’s wider environments come into play during the use of videoconferencing technologies. We describe the types of objects with which participants interacted (4.1) as well as the ways that they support the videoconferencing activities of people with cognitive concerns (4.2).

### 4.1 What Types of Objects Are Involved in a Videoconference?

In response to RQ1, which asks what types of objects participants interact with in the context of videoconferencing, we found a range of objects which include videoconferencing software and hardware necessary to run a videoconference, as well as objects beyond the videoconferencing interface. Table 3 summarizes the seven main categories and subcategories of objects from our analysis.

Participants engaged in videoconferencing through a range of *personal computing devices* including desktops, laptops, mobile devices, and tablets (*hardware*). Some participants used different devices depending on purpose or convenience (e.g., ”*if I’m not right by my machine, I might, but I am by a phone, I might pick up on the phone*.”). In terms of *software*, participants used a range of videoconferencing platforms (e.g., Zoom, Google Meet, Microsoft Teams). We found that most participants-initiated videoconferences were conducted on Zoom, while the platforms used for videoconferences they attended often depended on the organizing party (e.g., Echo’s physical therapy sessions were hosted on the software specified by her health insurance provider). Participants interacted with a wide range of videoconferencing-software features including video and audio settings, annotation tools, sharing screen features, and storage.

The above objects–and the interface in particular–are typically the focus of studies of videoconferencing in HCI. We also observed technologies that supported, but were not directly involved in running the videoconferencing. Echo used a *self-tracking device* alongside their videoconference, and Kai used their phone to support their communication on the call: “*I often see my cellphone as my second desktop. That way, I can look up company information on it*”. As might be expected, participants also used a range of peripherals and screens, including the touchscreen or other *input devices* such as keyboards and mice to interact with the screens of their devices. Some used *monitors* (and dual monitors in the case of Skyler) to get more space for the software they were using for or during the call. In terms of *visual and audio input*, participants used built-in visual (i.e., cameras) and audio tools, as well as speakers, soundbars, and headphones that participants connected to the device running the videoconferencing. TVs (Taylor), storage devices (flash drive, Phoenix), and printers (River, Morgan, Avery, Kai, Sage, Phoenix) were additional peripherals that some participants interacted with in the context of a videoconference, for example, to print items of relevance to an upcoming videoconferencing call.

**Figure 2:**
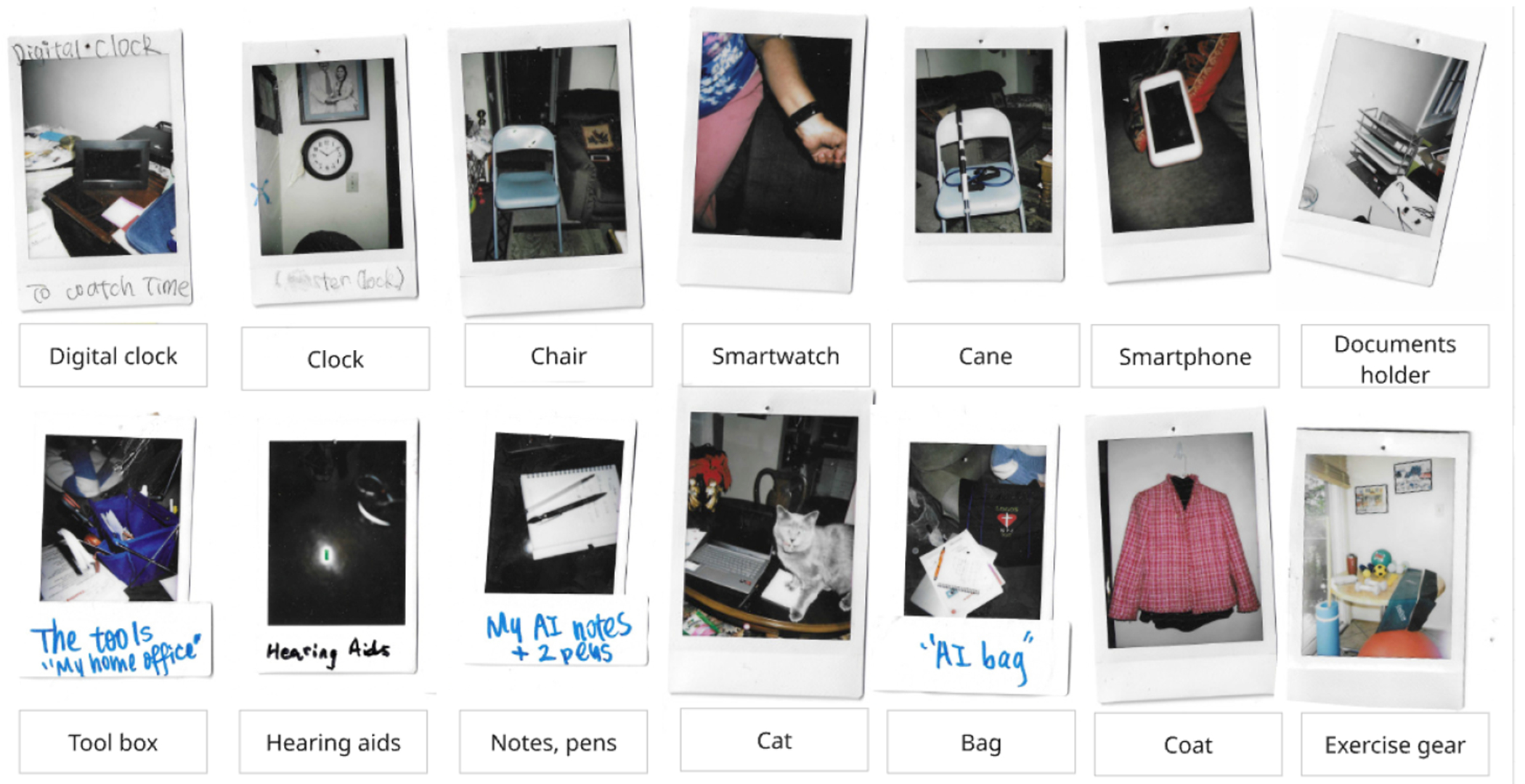
Examples of object types captured by participants through Polaroid photos during the diary study, spanning multiple categories beyond videoconferencing hardware and software, including other digital devices (e.g., digital clock, smartwatch), paper-based objects (e.g., notes), personal items (e.g., documents holder, bag, cane, hearing aids), built environment (e.g., chair, clock), and other (e.g., cat).

Participants also used a range of software outside of videoconferencing-specific software. This included digital clocks and calendars to orient themselves, as well as email, texts, and apps (e.g., WhatsApp, WeChat) to coordinate and access videoconferencing information and links. They used browsers for videoconferences but also to conduct activities concurrently with the call. They made notes through digital note-taking tools or take screenshots to capture information from videoconferences, and some utilize digital documents accompanying videoconferences (like the “ai handouts” captured on Taylor’s corkboard on Figure 2) or contribute to digital documents through presentation software (e.g., PowerPoint) or cloud-based documents.

In addition to the wide range of hardware and software described above, we categorized a large set of non-technological objects integral to use. Some of these served similar roles to digital tools, such as **paper-based objects**. Objects in this category, like their digital counterparts, supported scheduling, sorting, and retrieving information related to videoconferencing. This category predominantly consists of the *calendars* and *handwritten documents* that many participants used. Some of these objects require the use of technological objects for participants to produce them, such as printed handouts for physical therapy videoconferencing (Echo).

The objects above, while not always considered as an area in which HCI researchers can intervene, are still likely intuitive as objects that are involved in videoconferencing. We found a wide range of objects that, on first glance, seem further off. The **built environment**, or objects associated with the architecture of the space, played a major role in videoconferencing. This included structural designations such as *rooms*. Participants chose where to conduct their videoconferencing based on many factors within a room, such as whether a room had sufficient lighting (Skyler), was quiet enough (Morgan, Avery), was spacious enough for physical activities (Echo, Rowan), included furniture like a large desk for devices and work documents (Avery, Kai, Sage, Phoenix) or offered comfortable seating such as couch and or an armchair (River, Taylor). *Elements* of rooms such as walls, doors, and knobs also helped support specific activities on the videoconferencing (e.g., hanging resistance band on a doorknob (Echo)). *Decor* includes a range of functional and aesthetic items including clocks, fans, and lighting, which were specifically interacted with in the context of a videoconference. The built environment category also includes items that power or keeps other objects running during a videoconference, specifically, *internet, landlines*, and *power supply* (i.e., cords and chargers).

In addition to the often larger and immobile objects of the built environment, smaller and more mobile *personal items* were involved in videoconferences in various ways as well. Some objects such as glasses and hearing aids were *worn on the body* to make the videoconferencing and related documents or devices accessible (*a magnifying glass* serves a similar role for Rowan). Objects in this category also include the *ingestible* water or snacks that participants prepare in advance of (or sometimes after) a call, *writing tools* (pens and pencils), a *mat* that was placed under a mouse, and *bags* used as a container for other objects related to the videoconferencing. *Exercise equipment* also came up in this category, used during exercise videoconferences and including yoga balls, mats, resistance, bands and weights.

Nearby living beings served as an additional “special case” of objects (as they can be perceived by the senses). Animals and people were mentioned sometimes as introducing distractions that needed to be managed. Surprisingly, *weather* conditions (rain and “bad weather”) came up in a variety of ways, including leading people to do videoconferencing rather than meet in person, close shades, as well as affecting internet quality.

### 4.2 Objects support videoconferencing for people with cognitive concerns

RQ2 asked *how objects support videoconferencing for people with cognitive concerns*. We found that objects hold semantic and time-related information (4.2.1). Second, objects enable participants to distribute effort across time, both in preparing for a videoconference and activities after the call ends (4.2.2). Third, different affordances of different kinds of objects are leveraged by participants (4.2.3). We summarize the themes and subthemes of these findings in Table 4. We detail how these objects function to support the cognition of participants with cognitive concerns in each section after first providing context with the ways people experience and navigate memory and cognitive challenges in everyday life.

#### 4.2.1 Objects Hold Information.

Digital and physical objects carry information that is important for the call but difficult to recall without these supports, supporting participants by providing important semantic and time-related information.

##### Semantic Information.

Participants described challenges recalling *semantic information*, a term which refers to the correct words for people, objects, and concepts. Avery described a dinner with neighbors where: “*there were four or five times what, what I said, I wanna contribute to the conversation and tell you about, help me out, who’s a woman who wears the earrings all the time. And my neighbor told me*.” Similar challenges recalling names, faces, and content showed up in videoconferencing sessions. Participants used objects, and paper-based objects in particular, to support their ability to have semantic information available when needed.

**Figure 3:**
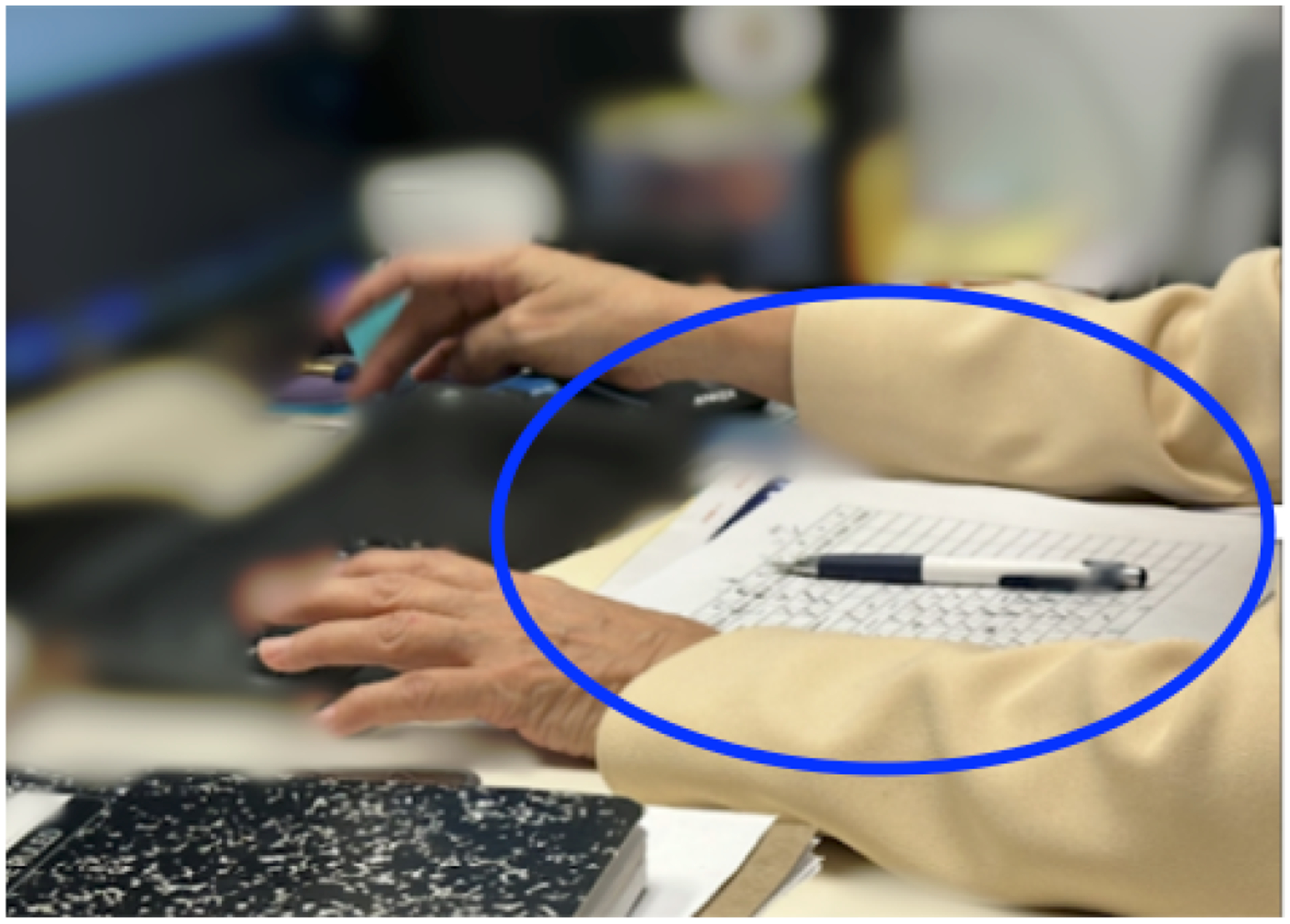
Phoenix has a printed sheet with attendee names for the videoconferencing session.

One example of a participant who used objects to hold semantic information is Phoenix, who continued teaching synchronous remote language classes as she experienced cognitive changes. She described variability in her ability to remember different kinds of semantic information: “*I remember numbers very clearly. With people’s names and faces I’m terrible, terrible, really terrible*…”^6^. When sharing her screen in class, when others interacted with her, only their faces appeared without names. She explained how in the past, when she could not recall a name in time or mistakenly called someone by the wrong name, she would be thrown off by the unexpected lapse, sometimes triggering other cognitive challenges such as forgetting what to say next. She now kept a paper-based roster at hand during videoconferencing (Figure 3). At the beginning of each class, she used it to check attendance and to familiarize herself with students’ names and faces. She then kept the roster nearby for the rest of the videoconference, referring to it whenever needed.

We also observed Avery using a sheet with attendees’ names, along with brief biographies: “*so I know who they are. Because I forgot them (in the past videoconferences)*.” Avery also printed and annotated reports that she had read but “*have absolutely no memory of (the contents)*” to bring to videoconferencing meetings. She kept these objects directly in front of her at the center of the table, within arm’s reach. During the videoconferencing, she reached for different documents without shifting her torso or turning her head, using her hands to sort through the pile and locate the needed sheet quickly.

##### Objects carry time-related information.

Participants described time-related information, such as dates and days of the week, as critical for everyday planning and scheduling. Several participants described challenges recalling this type of information. As Rowan explained, “*Sometimes I find myself asking, what day is it today?*”. In videoconferencing, people track start and end times for timely participation and consult dates and times for the planning and scheduling that can occur during a call. Issues with temporal recall led consequences, such as mistakenly scheduling multiple videoconferences at the same time (Phoenix).

**Figure 4:**
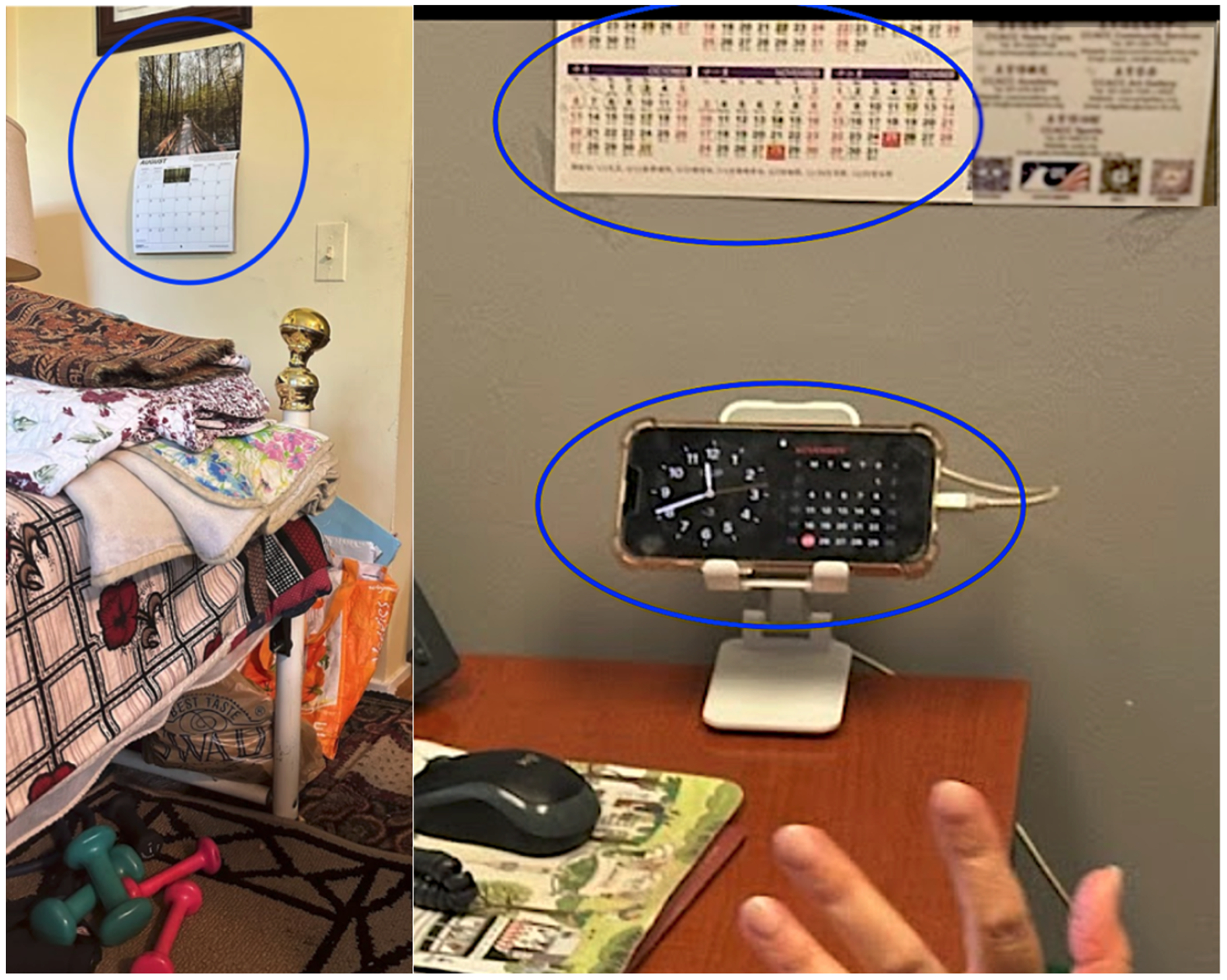
The left image shows a wall calendar next to Echo’s bed, which she consulted by walking a few steps before or during videoconferencing exercise. The right image shows Kai’s setup with a digital clock displayed on a phone and a paper calendar hung above on the wall.

Participants incorporated timekeeping objects to support temporal recall. When planning and scheduling became difficult, Avery reported changes in their ability to remember dates and commitments and treated the paper calendar as an additional memory source: “*I am accustomed to having my calendar in my head…now I have to rely more on the paper…but I still believe that it’s in my head*.” A nearby physical calendar functioned as an external memory resource that extended beyond videoconferencing for Echo and Kai (Figure 4).

**Figure 5:**
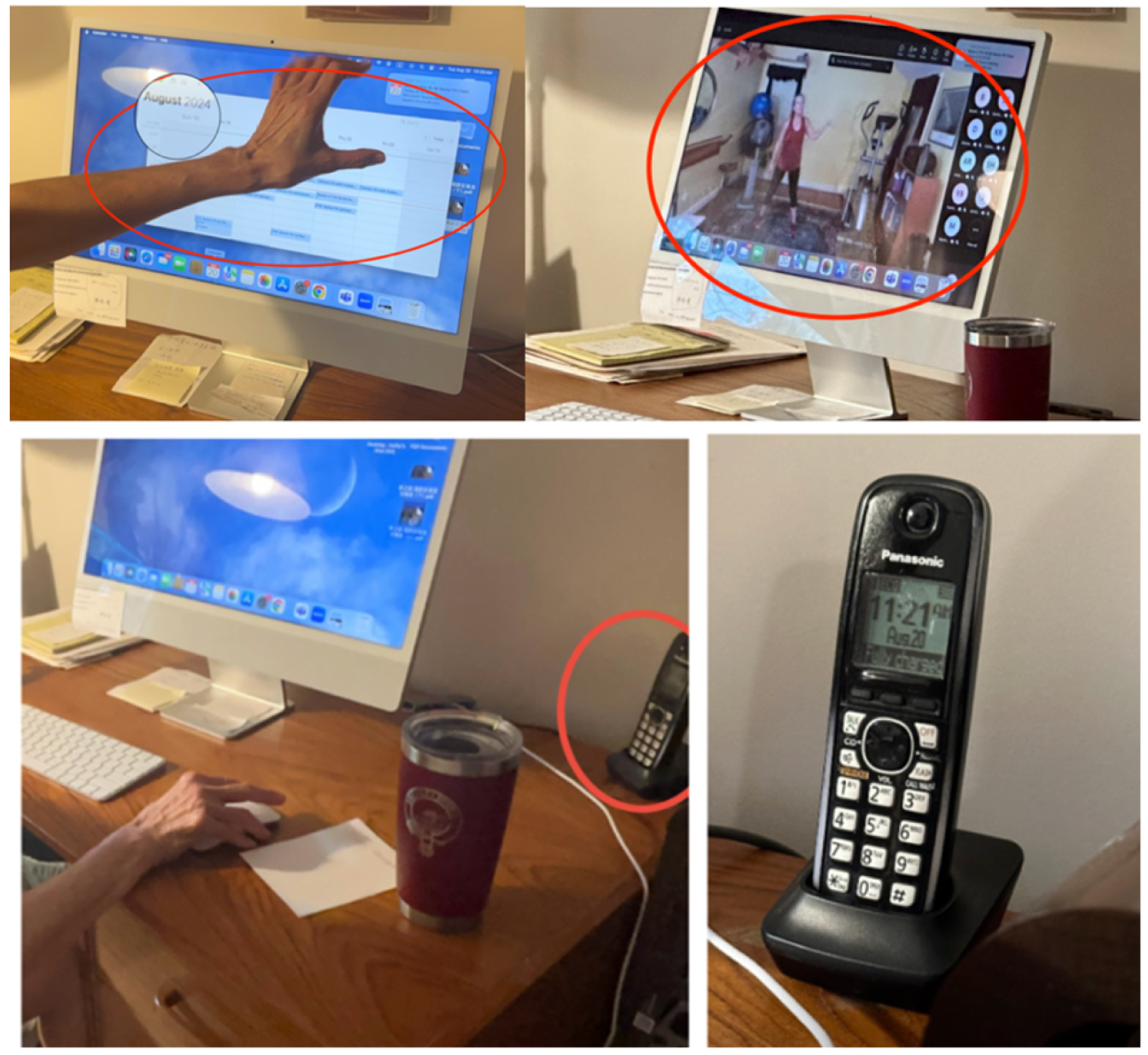
The top row shows the e-calendar Rowan consulted before the videoconferencing session, which became hidden once the videoconferencing platform took over the full screen. The bottom row shows the landline phone with a clock on Rowan’s desk.

This use of time-keeping objects did not mean that participants did not use digital time-keeping tools as well; the e-calendar, which displayed the time range of her daily schedule, reminded Rowan of videoconferencing session start times (see first image in the top row of Figure 5). Once Rowan enlarged her window in advance of the call, however, the e-calendar was no longer visible (second image in the top row of Figure 5). From that point on, the landline’s built-in clock (bottom row of Figure 5) became her reference for time. We observed her quick glances to the landline clock on multiple occasions: while waiting for the session to begin, she glanced at it and noted how many minutes were left; after the class, during brief chats with her instructor, she continued to check it to gauge how long she could remain on the call without making the instructor late for subsequent schedules.

#### 4.2.2 Objects Enable the Distribution of Effort Across Time.

Objects support participants in functioning cognitively at their best. They help participants distribute effort across time by preparing for videoconferences in advance and externalizing information during the videoconferencing itself.

##### Using objects to prepare.

**Figure 6:**
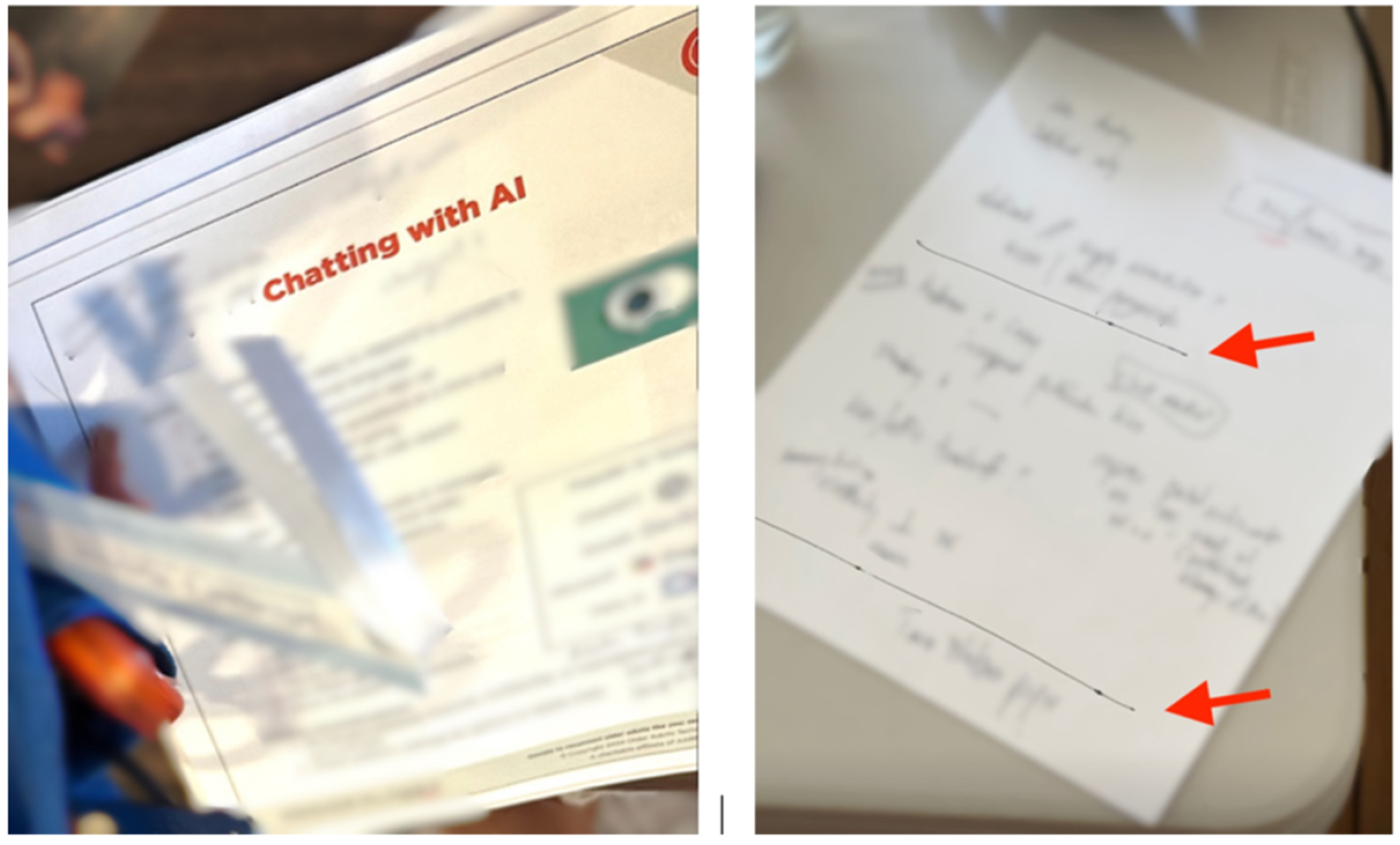
The left image shows a handout from Taylor’s videoconferencing AI class titled “Chatting with AI,” and the right image shows a paper note Skyler wrote during his work videoconferencing. The different sections correspond to preliminaries, logistics and minutes, and concepts to look up later.

Preparing ahead of time, such that tasks can “*be done whenever convenient, when there are not time pressures or other stresses*….” (page 4, [107]), helped lower cognitive load once participants were on a call. This was helpful for participants who had videoconferences occurring at times where they were not at their highest cognitive functioning. Kai’s videoconferences often occurred later in the day, and involved discussing daily plans. She created bullets in her journal with key points that she could use later in the day: “*In the early morning, when my mind is still relatively clear and before anyone comes to find me, I quickly write them down*.”^7^ She kept this journal within arm’s reach to support recall during the call. Preparing notes (Figure 6) in advance was an essential step for Taylor, who used them to refresh her memory before joining as part of a routine which she described as, “… *set everything up, see how much power I had left on the computer, reread the notes on how to get there (the AI tool used in class)*”.

##### Externalizing during videoconferencing through objects.

Cognitive capacity is limited. It is “*much harder to multitask*” (Avery) when engaged in communication during a videoconference. Nearby objects played a key role in capturing fleeting thoughts or information while participants engaged in a videoconference, helping them transfer ideas from their working memory to a more sustained external memory. Nearly all participants had note-taking tools like pens, scratch paper, book, and sticky notes within arm’s reach to be used during videoconferences. Even in spatially constrained setups, participants found ways to position these tools strategically, for example making space on a side table to hold notes (Taylor).

Constructing notes can offload the need to use additional cognitive capacity to later, such as ‘understanding’ some content during a call to a later time when cognition is not intensively engaged in videoconferencing. At times, Skyler had trouble understanding words related to “*high-level*” and “*facile*” concepts during videoconferencing meetings at work. “*I find that I’m just slower understanding what people are talking about generally*.” Skyler utilized the tactic of writing things down to support his cognition explaining that, “*I find it more likely that I remember things or understand things if I write them down*.” Skylar walked us through the notes that he took during videoconferencing: these included logistics as well as words such as the names of people and concepts (see between the two lines in Figure 6, redacted for confidentiality). These words were often annotated with question marks, and Skyler indicated that he planned to revisit them later to process them.

Objects also enable participants to save themselves effort later. Participants described times where they forgot plans or decisions shortly after making them, like scheduling lunch with someone but forgetting about it by noon (Phoenix). We observed participants markup to-dos at the videoconferencing when they already engaged in thinking and planning. For example, the last section on Skyler’s notes served as a to-do list that he developed as the meeting unfolded. He included the name of a document he planned to review in the following days given the topic of today’s videoconferencing meeting, to help him remember and follow through on tasks he needed to do later.

#### 4.2.3 Objects Enable the Distribution of Effort Over Space.

Researchers have noted that “*Space is a resource that must be managed, much like time, memory, and energy*.” (p190, [27]). Participants placed, stacked, hung, and grouped objects so that they could be accessed in a way that requires the least cognitive load. We identified three object affordances in our data that were leveraged by participants: grabbable, glanceable, and switchable.

##### Grabbable objects.

Grabbable objects are items placed within arm’s reach, which can be easily picked up during video calls. Hardware devices, peripherals (e.g., mice), and some paper-based objects were placed by participants on the desk or table where they were sitting and used to support videoconferencing tasks. Ingestible were also put within arms distance, enabling participants to satisfy hunger or thirst without getting up. Some objects were placed within arm’s reach on other surfaces like the floor or adjacent furniture when a fixed place was not preferred or ideal. This included personal items such as bags, exercise equipment, and a magnifying glass.

To explain why having objects within arm’s distance was so important, we start with an example from River’s everyday life. She had developed ways to cope with difficulties getting out of her house with all the items needed:

River had only just settled into her new residential community when the small frictions of daily life began to show. The drawers were different, the layout had changed, and familiar spots for everyday items were gone. Often, as she left home, she paused at the door and recited a four-syllable rhyme in her native language out loud: “身手要钱.” Each syllable cued a check on ID, phone, keys, wallet and turned a taxing memory task into a brief routine she could trust. The phrase had stuck with her for years because it also sounded like a common expression meaning “to reach out and ask for money,” which made it easy to remember.

Missing and searching for objects in the middle of videoconferencing can be particularly challenging as participants are occupied with other videoconferencing tasks or interactions. Taylor described searching for an object that she had forgotten to prepare in the middle of a videoconference call, only to experience a cognitive lapse while searching and forgetting which objects to search for. Avery also emphasized the importance of being able to grab objects quickly, explaining that if she did not take action right away, she would get “*confused*” later. Participants reserved time before the videoconferencing started to gather the objects they would need and place them within arm’s reach, like when River left a few minutes to grab her phone and glasses before the videoconference with her family started.

**Figure 7:**
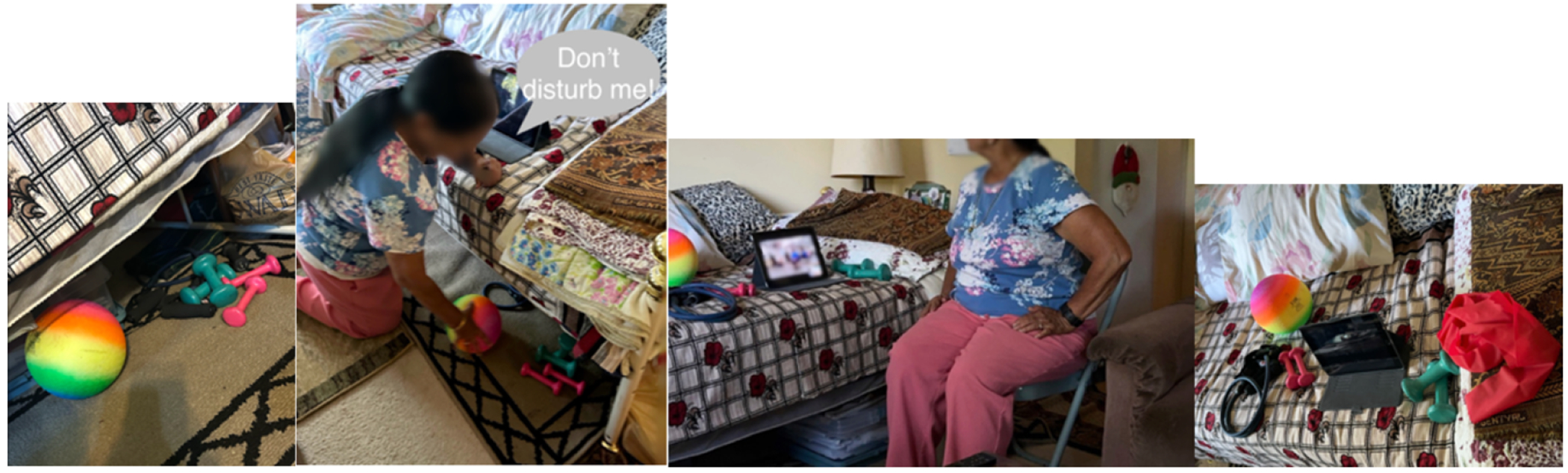
From left to right: exercise equipment stored under Echo’s bed; Echo grabbing the equipment before videoconferencing; Echo exercising during the videoconferencing session; and the layout of objects used for Echo’s videoconferencing exercise.

Designated spots stored groups of relevant videoconferencing objects. Echo’s videoconference on exercise ( Figure 7, image 3) can include different exercise gears (Figure 7, image 4) depending on the exercise instructor and their styles, sometimes weights, sometimes a yoga ball. Difficulty remembering objects was the most salient cognitive concern Echo described in her daily life, for example when she had recently forgotten to bring her wallet to the airport – again. When it came to videoconferencing, she had learned that the best way to avoid forgetting was to store all exercise equipment underneath the bed where she routinely joined videoconferencing sessions (Figure 7, image 1). It was evident how important getting objects into a place they were reachable was when, during the observation, the researcher asked her another question and Echo replied, “*Don’t disturb me*” as she focused on gathering all the objects from under the bed ten minutes before her videoconference began (Figure 7, image 2).

Some participants’ videoconferences required many objects. Sage carried chargers, a mouse and keyboard, headphones, external cameras, relevant printed or handwritten documents, and food or drinks, and stored these in a bag to bring from place to place without the need to recall and gather the things she needed every time. Taylor placed her “AI bag” (named by the theme of her videoconferencing) next to the chair before videoconference started, so that she could easily reach down, bend slightly, and grab what she needed for a videoconference. At the end of each videoconference, she put everything back: “*then I will put everything in my filing system, my bag, and that’ll be it*.” The analogy of the “*filing system*” shows how Taylor kept organized with preparation beforehand and wrapping up after videoconferencing, and she applied the same method to other kinds of videoconferences she attended (e.g., her Bible study group).

##### Glanceable objects.

Glanceable objects are items placed within the visual field. They are not the main focus during a videoconference, like the video feed often is, but they can provide important information through a quick eye movement. Glanceable objects are sometimes placed by participants in advance of a call, such as a calendar hung up on a nearby wall or post-it notes stuck to the desktop. They also may be already situated as a part of the larger plan of their living space, like a digital oven clock or a clock hung on the wall.

**Figure 8:**
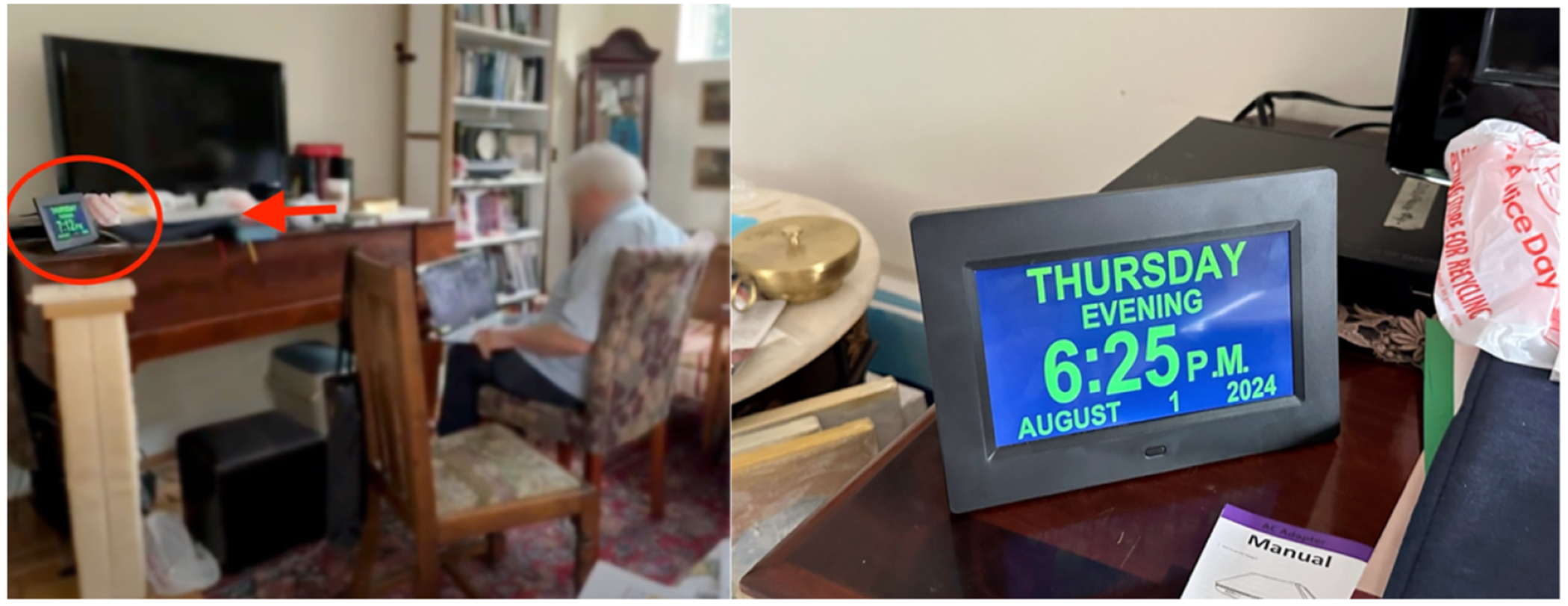
The left image shows Morgan’s videoconferencing setup with a digital clock on the desk, and the right image shows a closer view of the clock.

**Figure 9:**
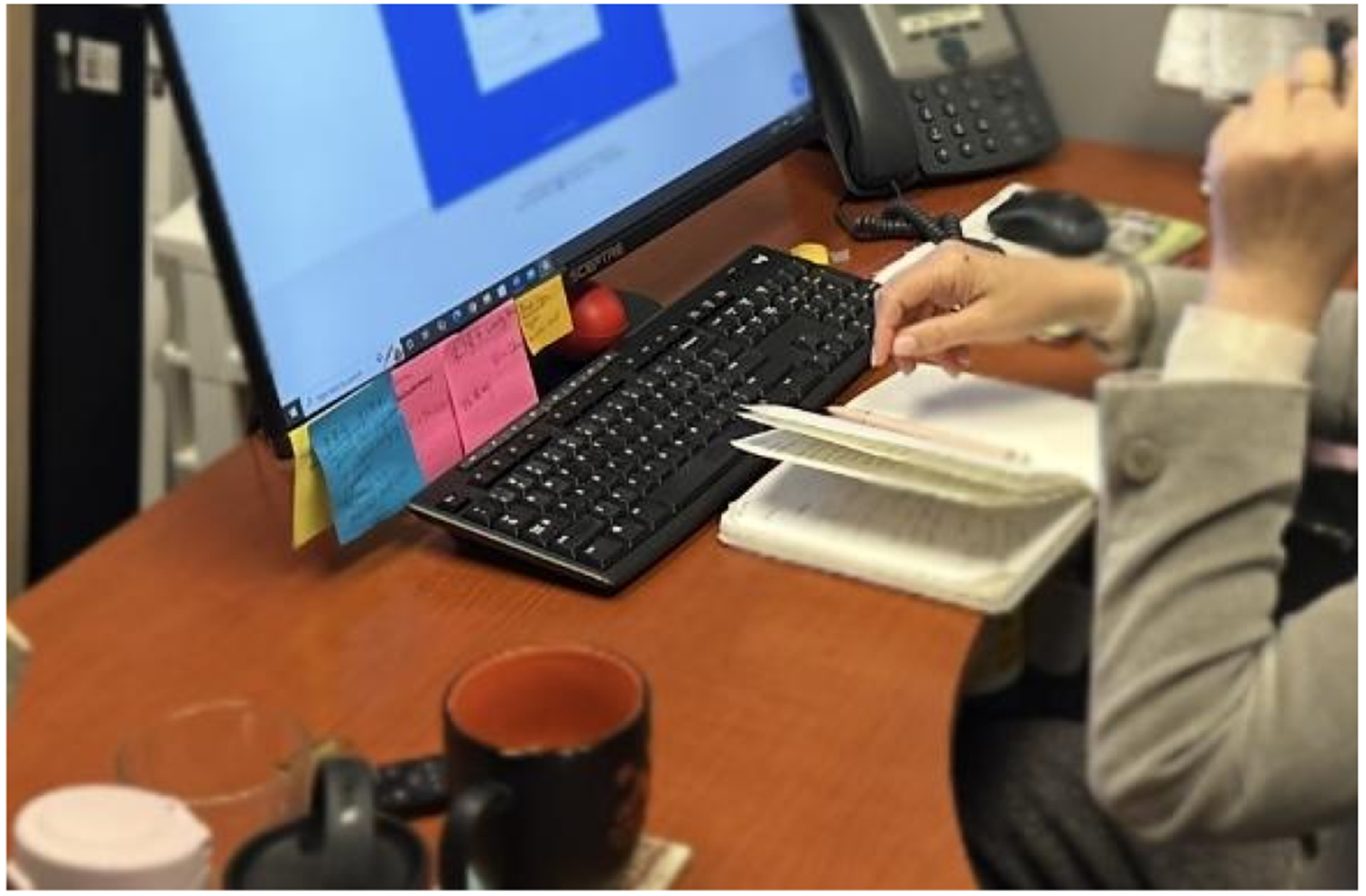
Kai positioned sticky notes on the edge of her computer screen, creating a glanceable space to support quick referencing without disrupting her main task.

In our observation of Kai, she scheduled her future videoconferences at the end of her videoconference call. She kept a paper wall calendar within her sightline (Figure 4) and took quick glances at it while continuing to speak during the call. These glanceable checks were especially helpful during the cognitive low period that can follow a long videoconferencing that she had. Having objects that carried temporal information nearby during scheduling came with an additional benefit: Phoenix, who also had a physical calendar sitting next to a desk, mentioned how it helped conceal their disorientation in front of their videoconferencing partners. Morgan did not have time-related difficulties but was prone to sensory distraction; he once experienced brief spatial disorientation of getting lost while walking in public noise. While videoconferencing, he selected the lowest-effort option: a quick head tilt to read the digital clock on his desk (Figure 8), then an immediate return to his history webinar. This glance avoided extra actions like grabbing his mobile phone, as he explained, “*it tells me when it is rather than looking at my cell phone or some other device*”. Other examples include the ways that participants use paper or sticky notes spread across surfaces (e.g., Kai, Figure 9): visible enough to prompt attention when needed, but not taking away from the main focus on the screen.

##### Switchable objects.

Switchable objects are items which lend themselves to having their physical state or status toggled or changed. This involves natural, low effort interactions for participants. For example, windows are opened and shut; lights are turned on and off; lipstick is put on or wiped off; objects are removed or brought to be in the room. Unlike grabbable and many glanceable objects, switchable objects could be at the periphery, such as windows or doors, or around the room, like ceiling light bulbs and fans. These objects could also be nearby, including those on the body, such as lipstick, earrings, workout clothes, and assistive devices like hearing aids (Morgan, Taylor). Participants often engaged with switchable objects, especially in advance of a call, to regulate their environment.

Participants used switchable objects to limit sensory distraction and support focus. Relevant switchable objects varied with the sensory focus participants prioritized for their videoconferencing. For instance, when listening in videoconferencing was the priority (“*I expected to listen more*”), Morgan would switch a variety of objects into different states to regulate auditory conditions: he closed the window to block out the rain sounds; he removed his cat from his living room so he did not hear his meow sounds^8^. Listening was a commonly prioritized sense: Phoenix shut the door to keep noises out of her room; Taylor turned offTV to focus on listening to less familiar content, “*it’s new stuff, no, I don’t turn on the TV because I have to concentrate*.” Visuals were another important sensory focus in videoconferencing. Previous work has discussed visual loss as common for people as they age such as the ability to select an object out of a busy environment [108]. Videoconferencing comes with a range of visual distractions people need to manage to support their focus. For example, Avery switched her videoconferencing desk to a tidy desk by “*straighten(ing) up the piles*,” which she described as supporting “*an orderly mind*.” Participants also switched on or off lighting sources such as desk lamps (Rowan, Avery, Sage, Phoenix, Taylor), ceiling lights (River, Morgan, Skyler, Echo, Rowan), or natural light through rotating the blinds (Avery).

A videoconference space can be an everyday living environment or an office area where objects not originally intended for videoconferencing are nearby (e.g., a TV and cats in the living room). Therefore, as switchable as these objects in a space, it does not always render out an ideal sensory environment for participants. When Taylor closed the window before her videoconference, she could still hear dogs barking or construction noise outside. When Kai closed the door before her videoconferencing, we observed a construction worker enter mid-call, and she immediately paused while speaking. This suggest that switchable objects can help people regulate their environments, but their effectiveness can be ultimately limited by the characteristics of the places where videoconferencing takes place.

## 5 Discussion

Our goal was to understand how elements in people’s wider environments come into play during the use of videoconferencing technologies. Towards this goal, we asked two research questions. **RQ1** asks what types of objects people interact with in the context of a videoconference. Our analysis revealed a range of objects involved in videoconferencing for ten participants with cognitive concerns (4.1). These included the hardware and software necessary to run a videoconference and technology used outside of the videoconferencing interface, as well as paper-based objects, personal items, objects in the built environment, animals, and weather. **RQ2** asks how these objects support videoconferencing for participants with cognitive concerns. We found that digital and physical objects beyond the interface play an important role in supporting videoconferencing. Drawing on distributed cognition, we found how three ways that these objects supporting cognition during technology use. First, we found objects hold information that is important for the call but difficult to recall without these supports, such as information about attendees or the date (4.2). Second, we found that objects also support participants in functioning cognitively at their best. They do this by allowing participants to leverage optimal cognitive functioning times ahead of a call to prepare and short-term memory during a call to document important items for later (4.3). Lastly, we found that objects enable the distribution of effort over space – participants leverage the affordances of grabbable, glanceable, and switchable objects to minimize cognitive load during a videoconference (4.4).

These insights point to design opportunities for researchers working with older people to focus on the work happening outside of today’s interfaces. We also discuss how the lens of distributed cognition could help us design better technologies to support cognitive functioning.

### 5.1 Design Implications: Opportunities Happening Outside of Today’s Interfaces

A research focus on objects is not novel, particularly in the context of designing for older people with cognitive impairment. Much past research has augmented or extended objects, for example to support social interaction [10, 31, 55, 84]. In this section of the discussion, we discuss design opportunities that exist when we *look at the ongoing work in which participants through objects*. We highlight three opportunities for design: 1) overlooked information needs, 2) overlooked effort, and 3) insights for remote synchronous communication. We engage with some of the technologies that researchers have focused on developing with older adults and people with cognitive impairments in the discussion below (e.g., banking technology, mobile shopping, network, AR, VR).

#### 5.1.1 Overlooked information needs in older adults’ technology use.

Our research adds to past work the importance of attending to objects *beyond the main interface* that are used for supportive tasks or to support access. Additional devices and software helped participants communicate outside of the videoconferencing platform and gather information, which reveals the need for a second information stream, or a parallel channel of support that supplements what the primary interface cannot provide. For example, participants who experienced cognitive concerns carried objects such as paper documents with annotations into videoconferencing to keep within arm’s reach for information that was otherwise hard to recall (e.g., names), or glanced at a clock next to the screen to check the time and date. These information streams (semantic and time-related) are salient in other everyday digital activities for general older adults. For instance, when engaging with banking technology, older adults might share their password with a close relative so that the close other can help them engage in financial services, such as withdrawing cash from an ATM bank [43]. Though HCI researchers are advancing interface design, *the information that flows alongside these digital activities is rarely considered in design*. One way to do so is to work with existing information sources (digital and physical) such as notes. Vines et al.’s study [85] with paper cheques with a group of people over the age of 80 shows that even seemingly non-tech objects can be made central to digital design. We can also design solely digital systems that can recognize and support multiple information streams, for example with multiple displays or connected devices.

#### 5.1.2 Overlooked efforts taken in older adults’ technology use.

Not recognizing the amount of work it takes to set up an effective videoconferencing outside of the steps of using an interface [36], we might assume that “*one-touch*” operation or simplified systems will solve the issues that participants experience. But finding and having exercise equipment nearby, tidying a desk, laying out notes around nearby surfaces, consulting with a calendar to stay mindful of the start time of videoconferencing were just a few examples of participant work that went beyond interacting with the videoconferencing device. A simplified interface does not support people with cognitive concerns in gathering all relevant tools and objects in advance. Lessening our focus on “*one-click*”, “*one-touch*” or simplified AgeTech systems for older people, and directing more effort to “*complement or add value to the familiar tools users already rely on*” [13] can help us avoid inadvertently disrupting existing, effective technology routines and environments [13, 30]. One promising example of this comes from mobile shopping services, where a study recommended integrating collaborative practices into shopping platforms after they found out that older adults actively consulted with family and friends about purchase plans when using these shopping tools [76].

Efforts participants put into videoconferencing can be prevalent across varying technologies and digital activities of daily living in supporting healthy aging. Mois and Rogers [65] identified that ensuring “*network access*” (e.g., Bluetooth, Wi-Fi, 3G/4G/5G) is often intertwined with applications of digital technologies such as cellular network connection that allows an individual to complete a digital activity such as creating an email account. And when network access is disturbed, it can have “*negative implications on the ability to complete digital activities*” [65, 109]. Moreover, establishing conditions that required less technical effort was also essential for specific types of videoconferencing purposes. For example, assembling exercise gear from underneath the bed was a necessary effort for Rowan to ensure she could grab anything she needed during her videoconferencing exercise. Studies of other emerging technologies with older adults reflect similar effort. For example, one study exploring the potential of augmented and virtual reality to promote wellbeing in older adults, suggested considering how the installation process can be simplified so that older adults can use it by themselves with less psychological burden [49].

#### 5.1.3 Insights for remote synchronous communication.

Remote synchronous communication has the potential to support older people’s ability to access opportunities from home including work, social interaction, healthcare, entertainment, and learning hobbies [15, 29, 32, 44, 62]. Insights from our work can complement research exploring remote synchronous communication. For example, researchers are supporting social activity in aging and dementia through VR [3, 26, 88]. Our work indicates that when we design systems without understanding the importance of physical objects (and those objects being in their right place), we might design VR interfaces that inadvertently remove access to exercise objects or the ability to orient oneself cognitively by glancing at a clock. In addition, findings from objects with “*switchable*” affordances are relevant to extended reality (XR) projects supporting synchronous communication. Consider one project that found that older participants enjoyed background sounds of social partners and wind moving through trees while playing Chinese chess in VR [88]. Our work indicates that for people with cognitive concerns, ambient noise and other sensory input should be easily “*switchable*” (i.e., not buried in menus) so that participants can regulate their environment in a way that works for them. Sometimes, hardware or tangible devices are necessary or more natural: past work has noted the importance of manually adjusting objects like self-facing cameras [15] or speaker volumes [29].

### 5.2 A Distributed Cognition Lens on Our Data

Leonardi et al. noted in 2009 that a fundamental challenge of designing home technologies for older people,

“… is to shed light on the complex net of interrelations exis(s)ting between daily activities, objects and meaning revolving around the home…. Addressing these research questions requires the simultaneous consideration of the physical and functional aspects related to living at home, of the representations and emotions associated to the domestic environment, as well as information about the everyday routines older adults living independently form and follow.” (P1703, [52])

Researchers have been working towards a deep understanding of how two of these factors connect, namely, how emotions and meaning connect to everyday routines and objects [10, 31, 52, 55, 98]. Less considered (though not entirely neglected [30, 41, 52]) is how older people’s “*physical and functional*” characteristics relate to the routines and objects that they have developed and use. We adopt the theoretical perspective of distributed cognition to fill this gap. This theory posits that, “*the organization of mind–both in development and in operation – is an emergent property of interactions among internal and external resources*.” (P177, [27]). In seeing cognition as emerging out of interactions between internal *and* external resources, people are not assumed to be self-sufficient to the degree that they could, for example, be put in an empty room in the middle of the night and defend their dissertation successfully. Rather, our internal cognitive functions (e.g., better or worse recall, anxiety tending to overwhelm our long-term memory and processing) work hand in hand with the resources we have at hand (e.g., detailed presentation notes prepared in advance). Like the social model of disability, this perspective allows us to consider how the environment enables or disables, such that disability is not seen as solely attributable to individual bodies [61]. Distributed cognition adds an understanding of how external resources specifically support cognition, which is well suited to the group we are working with.

Distributed cognition introduces the insight that: “Processes may be *distributed through time*^9^ in such a way that the products of earlier events can transform the nature of later events.” (p176, [27], emphasis ours). Past work describes large scale decisions made before the call such as family members [36] and older users [29] selecting devices or videoconferencing platforms. Our study reveals work that people with cognitive concerns are doing to support their cognition before a call, for example when Kai created bullet points in her journal early in the morning with a fresh mind and then brought the journal to her videoconferencing later that day. We also uncovered work done during the call to “transform the nature of later events” (p176, [27]), or events which take place after a call. This includes deferring tasks, such as Skyler noting down words and phrases that he found difficult to grasp in the meeting and returning to them after videoconferencing, and noting plans shortly after making them to jog memory after the videoconference.

When we design without accounting for the ways objects support people’s cognition across time, we will miss opportunities, for example to support participants when their cognition is *sharpest*. An AI notes feature embedded in videoconferencing platform does not capture “*planning*” that occurs in advance. We can look into efforts of HCI researchers who have explored ways to support capturing just-in-time information and retrieving it later [12, 35, 37, 40, 74] for specific contexts, reducing the effort needed to remember in the moment and still recall in the soon future. For instance, one project has used a large language mode (LLM) to infer the user’s memory needs in a conversational context, semantically search memories, and provide suggestions when users forgot something (e.g., a name just mentioned) during the conversation [90].

**Distribution across space** is another central concept in distributed cognition. Kirsh notes that “in having a body, we are spatially located creatures: we must always be facing in some direction, have only certain objects in view, be in reach of certain others. How we manage the space around us, then, is not an afterthought; it is an integral part of the way we think, plan and behave…” (p31, [38]). Kelly et al.’s discussion of videoconferencing frames distribution across space as a matter of maintaining connection and coordinating care at a distance, especially in aged care and transnational families [36]. Our work drills down into the immediate region of the user, showing how they actively manage the objects distributed within their environments to scaffold cognition through affordances (grabbable, glanceable, and switchable).

Kirsh explains that space can be used to simplify choice, whether by *highlighting* the ability to do some actions or *constraining* others [38]. We see choice simplification through *highlighting* when participants prepare drinks, paper, documents, and writing instruments to be nearby during a call. *Constraining* is evident when Avery cleaned off her desk to maintain an “*orderly*” mind. In terms of how this might translate into digital environments such as mixed reality, systems can *constrain* choice in space by determining how much information to display and where to display it based on users’ cognitive load [57].

A third tenant of distributed cognition is that cognitive labor can be **distributed across people** [33]. In narrowing our unit of analysis to people using videoconferencing and their material environment, we are missing the key role that other social partners play. Fortunately, the role of social partners in supporting technology use alongside older people with and without cognitive impairment is a topic that has been studied in detail in past work [47, 59, 71] including on synchronous remote communication with older adults [3, 36, 89].

Based on the above points and our read of the literature, we argue that a distributed cognition perspective is useful because it shows 1) the importance of the material environments that people configure to support their technology use; 2) how these environments support cognition, which is important for everyone but particularly salient in the context of cognitive concerns, and; 3) that people are making good decisions to take advantage of cognitive affordances that the material world offers but our flat digital screens do not.

We encourage future research to explore other, complementary theories in the vein of studying the context of technology use [68]. A continued focus on embodied interaction for those with cognitive concerns, which has allowed researchers to attend to the non-verbal interaction of people with more significant cognitive impairment [46], and might be useful for those with less advanced conditions as well. Situated action models [79] provide an additional promising theoretical foundation to study broader context of people with cognitive impairments, including artifacts like ones we found, but also individuals, and social groups in which they belong.

## 6 Conclusion

Elements beyond the interface have been making themselves known when more HCI scholars are turning to consider and explore the lifeworld and technology experiences of older adults. We studied videoconferencing use by ten older individuals with cognitive concerns in a week-long study of interviews, observations, and a modified diary study. Our work contributes to an understanding of how individuals with cognitive concerns can successfully engage in activity through videoconferencing. We do so by surfacing the role of some of the mundane, every day, non-technological objects that appear alongside more clearly relevant hardware and software in our analysis. Designing accessible software interfaces for older adults with cognitive concerns continues to be important. However, the environment around a technology warrants more attention than it receives. Our findings reveal opportunities to support the technology use of people with cognitive concerns by attending to objects and their affordances. We argue that a distributed cognition lens illuminates our data and helps us think about people with cognitive concerns differently.

## Figures and Tables

**Table 1:** Diary self-titles and example entries from participants’ modified diary study

Participant (pseudonym)	Self-titled diary	Example item from each participant
River	Documentary of Life^1^	type-II diabetes, ID, paper documents, iPad, glasses, volume control button, couch, ceiling light, magnifier
Morgan	Exploring the World Through the Web	laptop, charger (always on), printer, WIFI, cat, Gmail, lecture hall, YouTube, desk, clock on the desk
Skyler	Videoconferencing Life	monitor, laptop, mouse, headphones, office computer, ring lights, Zoom, worst chair, fans, shorts, logout, water
Echo	Let Us Meet Virtually	iPhone, chair for exercise, crutch, videoconferencing exercise container, Fitbit, notebook, dress, sister
Rowan	Fitness Fun!	iPhone, laptop (iMac), study room, sports shoes, keyboard, camera, yoga balls, weights, curtains, slides, desk, chair, cushion, lamps, lights, walls
Avery	Videoconferencing in Context	desktop computer, the messy desk, lighting, glasses, printouts, speaker, earrings, pens, TV, office, background, the dog, bed, Todo list, sticky notes
Kai	Discussion of [videoconferencing meeting topic]	desktop computer, second laptop (mobile phone), paintings in the background, physical calendar, water, coffee, snacks, headphones, external camera, office, landline
Sage	Readiness for 1-1	laptop, mouse (external), charger, glasses, Gmail, clock, ChatGPT, personal computer, notes, a bottle of water
Phoenix	Tuesday [videoconferencing class name] Preparation	phone, folders, smart brain, annotation tools, textbooks, the door, emails, fresh memory, desktop, paper agenda, attendee roster
Taylor	AI Wed Meeting	my AI notes, AI handouts, the tools (my home office), TV, glasses, charger, hearing aid, cup of tea

**Table 2:** Participants’ Demographics and Cognitive Concerns

Participant(pseudonym)	Age Range	Race/Ethnicity	Type of Cognitive Concerns	Number of Year Experienced	Cognitive Concerns	Additional Disability/Accessibility Information
River	70s	Asian	SCD	2	recalling names (of people who sees everyday), remembering locations of everyday objects (e.g., keys, wallet)	vision (need to wear glasses when reading her iPad screen)
Morgan	80s	White	SCD	2	navigating new physical locations; recalling names; finding the exact phrases or words	hearing (wearing cochlear implants for amplified sound)
Skyler	60s	White	SCD	2	finding the exact phrases or words; monitoring progress of everyday task (e.g., left the drawer open); retaining semantic information (e.g., names)	hearing (“sometimes I have a hearing problem.”)
Echo	80s	Asian	SCD	2	recalling names; occasionally forgetting bringing things (such as personal checks)	mobility challenges at the time during the study (using a crutch to move around)
Rowan	70s	Asian	SCD	2	navigating new physical locations; wayfinding; remembering locations of everyday objects; retaining short memory	N/A
Avery	70s	White	MCI	10	recalling names; navigating new physical locations; retaining information over a week	mobility (“complicated to travel outside of where you [Avery] live”)
Kai	60s	Asian	SCD	2	attention management (e.g., stay focused), retaining information for a long time	N/A
Sage	50s	Asian	SCD	5	finding the exact phrases or words; recalling autobiographical data (e.g., placed travelled); recognizing familiar faces (e.g., colleagues); multitasking (e.g., cooking several dishes at the same time)	N/A
Phoenix	60s	Asian	SCD	10	recalling names, numbers; recognizing familiar faces (e.g., neighbors); retaining temporal information (e.g., dates); navigating new physical locations	N/A
Taylor	70s	White	SCD	7	retaining information (e.g., remembering chords); remembering locations of everyday objects (e.g., cell phone); multitasking (e.g., cooking several dishes); navigating different locations (e.g., rooms within home); recalling names	hearing, depression (depression occurring while grieving a family member’s loss)

**Table 3:** Types of objects participants with cognitive concerns interact with in the context of a video call.

Main Category	Subcategory	Examples
Hardware	computing devices peripherals & screens	personal computing devices, tablets, mobile devices, and self-tracking device (Fitbit) flash drive; keyboard; mouse; dual monitors; screen; printer; TV; speaker; headphones; cameras
Software & Web Platforms	VCs software, features & UI non-VCs software, features & UI	sharing screen feature; video & audio on/off feature; chat; VC participants list; self-video feed; other VC attendees’ video feed Word; ChatGPT; Google; emails; texts, digital alarms; e-calendar
Paper-based Objects	identification documents handwritten documents calendars printed documents	ID; library card to-do list; sticky notes; notes on paper; diary physical calendars printouts; paper documents e.g., attendees sheet/roster; ”my ai notes”; printed text/images; class agenda
Personal Items	body-worn items ingestible exercise equipment bags writing tools magnifying glass mouse mat	hearing aids; glasses; clothing; makeup; jewelry; sneakers; socks; shorts; lipstick; earrings water; coffee; “cup of tea”; snacks; caffeine yoga balls; yoga mat; resistance bands; weights bag, purse pens, pencil magnifier “mat”
Built Environment	rooms structural elements furniture decor power supply internet landline for phone	living room with paintings surrounded by; bedroom; office walls; the door; the doorknob in p4’s home couch; desks; chair; cushion; bed; bookshelf curtains; clock; fans; lamps charger; electric cords WIFI; internet landline
Other	pets and animals people weather	cat; dog; birds brother; husband; relatives; wife; kids storm; rainy weather; rain and shine

**Table 4:** Summary of themes and subthemes describing how objects support videoconferencing for participants with cognitive concerns.

Themes	Subthemes	Example
Objects Hold Information	Objects hold semantic information Objects carry time-related information	Phoenix gazed at the paper-based roster throughout the videoconference to remember people’s names. Echo set up a physical calendar beside her videoconference spot to remember dates.
Objects Enable the Distribution of Effort Across Time	Preparing ahead of videoconferencing through objects Externalizing during videoconferencing through objects	Kai wrote in her journal early in the day when her cognition was better so she could bring to the videoconference later. Taylor jotted down fleeting thoughts during videoconferences and revisited them later.
Objects Enable the Distribution of Effort Over Space	Grabbable objects Glanceable objects Switchable objects	River grabbed her phone and glasses nearby before the videoconferencing started. Kai glanced at the Post-it notes hung on her computer for quick checks during the videoconference. Taylor turned off the TV to better focus on listening during the videoconference.

## CCS Concepts

• Human-centered computing; • Human-Computer Interaction (HCI).; • Age;; • Seniors;; • People with disabilities;;

## Footnotes

2 Subjective Cognitive Decline (SCD) [73]: an individual’s self-reported experience of worsening memory or other cognitive abilities compared to their previous functioning, despite the absence of neuropsychological assessments

3 Mild Cognitive Impairment (MCI) [93]: a diagnosis that notes the presence of some cognitive decline, such as difficulties with memory, language, or spatial abilities. For some, MCI can be a precedent stage to dementia, but for others, it can resolve (e.g., with treatment for depression or medication changes).

4 “Dementia is the loss of cognitive functioning – thinking, remembering, and reasoning – to such an extent that it interferes with a person’s daily life and activities.” [94].

5 While the concept of software, features, or UI as “digital objects” may seem strange at first, as Dourish and Mazmanian point out, there are existing theories which understand that “the ineffable stuff” of digital abstractions is encountered only ever in material forms” (page 8, [92]). For the purposes of this study, this means that software and web platforms meet our definition of an object because people interact with them through their senses (typically “seeing” or “hearing” information on the interface).

6 Quote in participant’s original language: “我对数字很记得很清。我对人名, 我对这个脸很糟糕,糟糕,很糟糕…”

7 Quote in participant’s original language: “我这个(journal) 是我天天会带着的…就是一早上来的时候头脑还比较清楚还没有人来找我我赶快把它写下来…”

8 In a playful nod to Schrödinger’s cat, the participant’s cat may have continued meowing elsewhere, yet once removed from Morgan’s videoconferencing space, it became silent for the participant.

9 Kelly et al. similarly organize a discussion section around how the work of video calling as distributed across time, space, and people [36]. Our work aligns with and extends their findings as described.

## A Apendix

### A.1 Demographics Questions

I. General

1. How old were you at your last birthday?
2. What is your gender?
3. How would you describe your race or ethnicity?
4. What is the highest degree or level of school you have completed?
5. Is there any engaged partner you would like to participate in the study with you? If yes, would it be acceptable for us to contact them to obtain their consent for participation?

II. Memory or other cognitive concerns

We would like to learn more about your concerns about your memory or the way you are thinking.

At the screening, you mentioned that you have (dementia, MCI, or SCD), is that correct?

Part 1. Pre-clinical Questions^1^ (More Open-Ended):

If has dementia or MCI:

*For people with dementia:* Can you share the diagnosis that you have?_____

*For both groups:* When did you receive this diagnosis?_____

**Table:**

Onset and Duration
What did you first notice that made you think you might have memory or other cognitive concerns? When did you first notice [the symptoms they mention or general memory or cognitive decline?]
Interventions and Support
Have you consulted a healthcare professional about your cognitive concerns? What did they recommend? Do you have a support network (family, friends, caregivers) that assists you with memory or cognitive-related tasks?
Part 2. Clinical Questions^2^ (4A’s+ Oriented):
Amnesia-Related
Do you sometimes find it hard to remember things? Can you provide an example? How frequent are these instances? (Some examples include what you read on TV, important dates or facts like your birth date or a well-known address, your kids’ birthday, the name of your previous employer.)
Aphasia-Related
Do you ever find it hard to remember or understand words when speaking with others? Could you describe a situation when this happened?
Agnosia-Related
Do you have difficulty recognizing familiar objects, faces of relatives, or familiar settings? Can you share an instance of this?
Apraxia-Related
Do you have difficulty navigating any physical space or environment? Can you describe a specific experience? (Some examples include using utensils to eat, inability to write, buttoning a shirt, zipping a zipper, or putting one foot in front of the other. Note: These examples are important to help participants differentiate Apraxia related symptoms from muscle ability.)
Executive Functioning-Related
Do you have difficulty managing and planning activities? Could you give an example of when this has been challenging? (Some examples include going to a supermarket without a shopping list and being unsure if you bought everything you wanted, have difficulty planning a Thanksgiving dinner, having trouble balancing your checklist, initiating activities, or planning a trip.)

1 Jessen, F., Amariglio, R. E., van Boxtel, M., Breteler, M., Ceccaldi, M., Chételat, G., … & Subjective Cognitive Decline Initiative (SCD-I) Working Group. (2014). A conceptual framework for research on subjective cognitive decline in preclinical Alzheimer’s disease. Alzheimer’s & Dementia, 10(6), 844–852. https://doi.org/10.1016/j.jalz.2014.01.001

2 Alzheimer’s disease. UCHealth. Retrieved June 25, 2024, from https://www.uchealth.org/diseases-conditions/alzheimers-disease/

### A.2 General information about participants videoconferencing use

**Table:**

Participant (pseudonym) River	Videoconferencing Devices (s) tablet (always)	Type of videoconferencing we Observed familial communication	Videoconferencing Platforms Used WeChat (only)
Morgan	laptop (always)	online cultural seminars	Zoom, Google Meet
Skyler	laptop, mobile phone (occasionally), desktop computer	professional work meetings	Microsoft Teams, Zoom
Echo	tablet, mobile phone (occasionally)	virtual exercise session	WhatsApp, Microsoft Teams, Zoom
Rowan	laptop, mobile phone	virtual exercise session	Google Meet, Microsoft Teams
Avery	desktop computer	professional work meetings	Zoom
Kai	laptop, mobile phone	online discussion groups	Zoom
Sage	desktop computer	professional work meetings	Zoom
Phoenix	laptop	teaching language classes	Zoom
Taylor	laptop (always)	attending AI-focused classes	Zoom
